# Qualitative Exploration of Health Care Professionals’ Experiences Caring for Young People With Acute Severe Behavioral Disturbance in the Acute Care Setting

**DOI:** 10.1016/j.acepjo.2024.100030

**Published:** 2025-01-13

**Authors:** Elyssia M. Bourke, Ned Douglas, Ziad Nehme, Jonathan Knott, Simon S. Craig, Franz E. Babl, Pediatric Research in Emergency Departments International Collaborative (PREDICT) research network

**Affiliations:** 1 Emergency Research Group, Murdoch Children’s Research Institute, Melbourne, Victoria, Australia; 2 Department of Critical Care, University of Melbourne, Melbourne, Victoria, Australia; 3 Grampians Health, Ballarat, Victoria, Australia; 4 Royal Melbourne Hospital, Melbourne, Victoria, Australia; 5 Department of Pediatrics, University of Melbourne, Melbourne, Victoria, Australia; 6 Centre for Research and Evaluation, Ambulance Victoria, Melbourne, Victoria, Australia; 7 School of Public Health and Preventive Medicine, Monash University, Melbourne, Victoria, Australia; 8 Department of Pediatrics, Monash University, Melbourne, Victoria, Australia; 9 Emergency Department, Monash Medical Centre, Melbourne, Victoria, Australia; 10 Emergency Department, Royal Children’s Hospital, Melbourne, Victoria, Australia

**Keywords:** Pediatrics, acute severe behavioral disturbance, qualitative study

## Abstract

**Objectives:**

To describe the experience of health care professionals involved in the care of young people with acute severe behavioral disturbance across the acute care setting.

**Methods:**

We used purposive and snowball sampling to recruit paramedics, nurses, doctors, and mental health clinicians caring for young people with acute severe behavioral disturbance in the prehospital and/or emergency department environments. We conducted one-to-one telephone-based semistructured qualitative interviews with each staff member. The audio recordings were transcribed verbatim, and participant pseudonyms were assigned. We iteratively developed a thematic coding structure. Data collection continued until thematic saturation was reached.

**Results:**

We interviewed 31 health care professionals—12 doctors, 5 nurses, 7 mental health clinicians, and 7 paramedics. Participants outlined factors they felt contributed to the young person’s behavioral disturbance. They detailed the management strategies used. Participants spoke about their exposure to physical violence while managing these young people and the challenges of balancing patient and staff safety. There was a significant personal impact on participants through providing care to this cohort. Participants acknowledged the workflow, staff resource, and bystander impacts of these presentations.

**Conclusion:**

Based on participant’s experiences, health care staff aim to provide high-quality care to young people with behavioral disturbance in circumstances that present risks to their safety. There is variability in the way staff are currently managing these young people likely because of the limited high-quality evidence currently available, highlighting key areas for future research.

**The Bottom Line**

This qualitative study describes the experience of health care professionals caring for young people with acute severe behavioral disturbance. Thirty-one paramedics, doctors, nurses, and mental health clinicians were interviewed. Management varied considerably between staff likely due to lack of available high-quality evidence. Staff reported occupational violence as being a challenge when caring for these young people and feelings of frustration, hopelessness, helplessness, and sadness were voiced after providing care to this cohort. Despite these challenges, these health care professionals aimed to provide high-quality care to these patients while balancing the safety of the patient, staff, and bystanders.

## 1 Introduction

### 1.1 Background

Pediatric acute severe behavioral disturbance is a common presentation in the acute care setting.^1^, ^2^, ^3^ This high-risk condition caused by profound emotional dysregulation presents considerable physical and psychological risk to the young person and health care staff caring for them.^4^ Because of the urgent need to achieve behavioral containment in these instances, care is often commenced in the prehospital setting^5^ before the young person is transported to the emergency department (ED) for their ongoing management.

### 1.2 Importance

A variety of nonpharmacologic and pharmacologic strategies are utilized in the care of these young people.^6^, ^7^, ^8^ These strategies are instituted by a range of health care professionals including paramedics, doctors, nurses, and mental health clinicians. Currently, there is limited high-quality evidence regarding the effectiveness of these methods.^9^ To bridge this evidence gap, a detailed understanding of the experiences of health care staff working in the prehospital and ED setting is required, with a particular focus on the current management strategies being utilized during the care of these young people.

### 1.3 Goals of This Investigation

The goals of this study were to understand the experiences of health care professionals involved in the care of young people with acute severe behavioral disturbance across the acute care setting and determine the challenges faced by these staff to guide the development of future research priorities.

## 2 Methods

### 2.1 Study Design and Setting

This qualitative study consisted of semistructured telephone interviews with staff involved in the care of young people presenting with acute severe behavioral disturbance in the prehospital and ED settings. We developed the interview guide (Appendix A) with input from previous literature^3^^,^^10^^,^^11^ and a discussion among all of the study authors. The guide was intended to be broad and covered topics including the participant’s general experiences managing these young people. Participants were afforded the opportunity to provide any additional or clarifying information that they thought would be relevant. The interview guide was not piloted before study commencement, but when new topics arose in the interviews, these were added to the interview guide in an iterative manner. We have used Consolidated Criteria for Reporting Qualitative Research (COREQ)^12^ to report our methods and results. The Royal Children’s Hospital Human Research Ethics Committee approved the study (RCHM-2020-203503) and governance approval was obtained from Ambulance Victoria (R20-023).

#### 2.2.1 Setting

This study was undertaken across the prehospital sector and EDs in Australia. ED staff (medical and nursing staff and mental health clinicians) were recruited across regional and tertiary EDs from 6 Australian states and territories. Paramedics were recruited from one Australian state where they worked in regional and tertiary settings.

#### 2.2.2 Selection of participants

Participants were contacted using a combination of purposive and snowball sampling. Each health professional group was recruited separately. The doctors who participated were able to express their interest in being involved after completing a separate but linked survey of pediatric behavioral disturbance undertaken across Australia.^10^ The mental health clinicians were invited to participate by email. Paramedics were engaged through internal communications of the state ambulance service. Nursing staff were contacted through purposive and snowball sampling. This included direct email contact with nursing staff who were the nominated mental health leads for their EDs and through nursing staff or medical staff suggesting nurses within their department who may be interested. All participants were provided with the Participant Information and Consent form to review before confirmation of participation. No reimbursement was provided.

#### 2.2.3 Research team

The research team consisted of the principal investigator and interviewer who was an emergency physician and PhD student (EMB). She had no personal relationship with any participants but did have a working relationship with a number of the participants. Although no specific training was provided to EMB in relation to this project, she has previous experience with qualitative interviewing and software.^13^ EMB acted as the primary coder, with ND a retrieval physician and clinician researcher acting as the secondary coder. Both EMB and ND have a pre-existing research interest in the management of pediatric behavioral disturbance.^5^ Three researchers (FEB, JK, and SC) were involved in contributing to the study and interview guide design, data collection framework, analytic framework, and the review of manuscript drafts. ZN, a paramedic and research lead in the state ambulance service, was involved in governance approvals, participant recruitment, and review of the protocol, interview guide, and manuscript drafts.

#### 2.2.4 Measurements

One interviewer (EMB) conducted all interviews by phone. The participants were therefore able to undertake the interview in a location of their choice including their workplace or residence. An open-ended question regarding the participant’s experience managing young people with acute severe behavioral disturbance was used to initiate the discussion, which was followed by a number of prompts used as required to cover the key topics of interest.

Interview length ranged from 26 to 73 minutes. The interviews were audiotaped and professionally transcribed verbatim. All identifiers were removed at this time. Pseudonyms have been used in place of the participants’ real names and epithets attached to denote the participants’ professional group. All transcripts were subsequently reviewed by EMB for completeness while listening to the audio recording to ensure accuracy. Transcripts were not reviewed or corrected by participants before analysis. No field notes were made during or after the interview.

### 2.3 Data Analyses

Data were imported into and coded using the software *NVivo 14* (QSR International)^14^ by the primary coder (EMB). Transcripts were analyzed using a constant comparative approach to generate themes.^15^ Practically, this involved grouping raw data into initial concepts, which were then further classified into overarching themes through an iterative process of comparing the data. The secondary coder (ND) independently reviewed a random selection of 20% of the uncoded transcripts to confirm coding consistency. Because each professional group was interviewed in sequence, interviews continued until thematic saturation was achieved, defined as no new themes emerging across or within the professional group for 3 consecutive interviews.

After analysis, a codebook (Appendix B) was iteratively developed by the primary (EMB) and secondary (ND) coders and reviewed by all remaining authors (FEB, JK, SC, and ZN).

## 3 Results

### 3.2 Interviews and Transcripts

We conducted interviews with 31 health care professionals involved in the care of young people with acute severe behavioral disturbance in Australia. We identified 60 codes, which are presented in the form of a coding tree (Fig) and a codebook (Appendix B).

**Figure:**
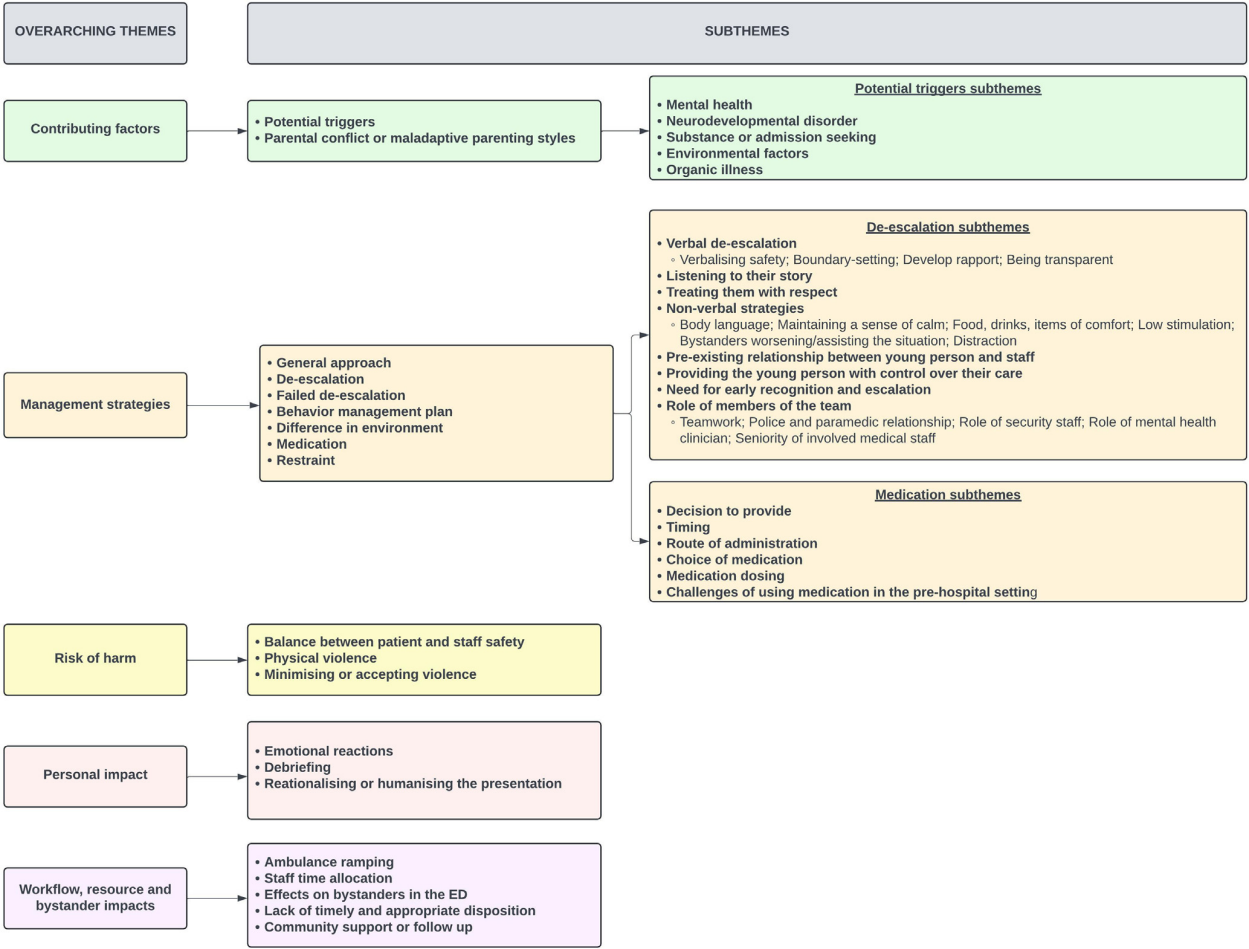
Key themes and subthemes.

#### 3.2.1 Characteristics of study participants

The participants included 12 doctors, 5 nurses, 7 mental health clinicians, and 7 paramedics. These individuals worked across prehospital and ED settings in 6 Australian states and territories. Rural, urban, and tertiary ED settings were included. Table 1 provides details as to the participant and interview characteristics.

**Table 1:** Demographic characteristics of the interviewees.

Participant pseudonym	Gender	Professional background	Duration of interview (h:m)
Kate	F	Doctor	00:26
Julie	F	Doctor	00:34
Paul	M	Doctor	00:55
Joseph	M	Doctor	00:37
Sally	F	Doctor	00:34
Harry	M	Doctor	00:43
Arthur	M	Doctor	00:35
Sam	M	Doctor	00:52
Chris	M	Doctor	00:51
Ben	M	Doctor	01:02
Nicole	F	Doctor	00:47
Betty	F	Doctor	01:13
Carmel	F	Mental Health Clinician	00:45
Judy	F	Mental Health Clinician	00:26
Beth	F	Mental Health Clinician	00:39
Anthony	M	Mental Health Clinician	00:44
Kim	F	Mental Health Clinician	00:49
Angela	F	Mental Health Clinician	00:29
Cassandra	F	Mental Health Clinician	00:23
Mary	F	Paramedic	01:01
Pamela	F	Paramedic	00:52
Peter	M	Paramedic	01:05
Chloe	F	Paramedic	00:55
John	M	Paramedic	00:48
Mark	M	Paramedic	01:06
Nathan	M	Paramedic	01:02
Patti	F	Nurse	00:32
Annabelle	F	Nurse	00:30
Hazel	F	Nurse	00:35
Harriet	F	Nurse	00:36
Charlie	M	Nurse	00:34

F, female; h:m, hours:minutes; M, male.

Throughout the interviews, health care professionals discussed a number of key themes including factors contributing to the development of acute behavioral disturbance; management strategies; risk of harm; the personal impact of these presentations; and workflow, resource, and bystander impacts. These themes are summarized with key quotes provided to highlight the participant’s experiences. The overall emergency physician experience is also specifically highlighted in Table S1.

##### 3.2.1.1 Contributing factors

Participants outlined the potential triggers they felt had contributed to young people experiencing an episode of acute severe behavioral disturbance (Table 2). Mental health conditions were viewed as a common trigger. Participants highlighted young people with a history of neurodevelopmental disorders, in particular those with autism spectrum disorder, as having a preponderance for experiencing acute severe behavioral disturbance. Environmental factors within the acute care setting were acknowledged as having the potential to worsen or improve the behavioral disturbance. Participants emphasized how conflict between the parent and child often escalated the situation. Maladaptive parenting styles were viewed as a contributor to the development of acute severe behavioral disturbance in some instances.

**Table 2:** Contributing factors.

Contributing factors
*The most recent child…came in as a 17-year-old who is in that horrible prodrome of schizophreniform illness…his violence, which is dissociative, it’s almost like a guarded dissociative state where he seemed to randomly just go completely insane and want to hurt anyone that was near him and smash everything* (Sam, Doctor)
*The kids with autism…or other intellectual disability whose aggression is their way of expressing frustration…I think the worst behavioral patients I’ve looked after fit into this category…[the parents] get to the point where they just say “my kid is now 15 and he’s too big, I can’t restrain him…he’s breaking all my walls”* (Harry, Doctor)
*One thing that I notice escalates…kids…are long waits…it’s the nature of the emergency department, but it’s hard to watch someone escalate purely just because they’re frustrated on waiting* (Harriet, Nurse)

##### 3.2.1.2 Management strategies

The participants spoke in detail about their approach to managing these young people (Table 3). Overall, there was a focus on balancing the rights of the young person against their safety as well as the safety of staff and bystanders.

**Table 3:** Management strategies.

De-escalation strategies
*You need to know how to be able to talk to them. Doctors who are very doctor-like and very prim and proper and talk the medical language, they’re not going to get through to a 15-year-old that’s going off* (Ben, Doctor)
*I tend to be pretty relaxed…I sit down and I usually just have a social chat if I can first to get a feel for what’s going on…try and make them as comfortable as I can* (Sally, Doctor)
*I sit on the end of the bed. I introduce myself. I tell them I’m here to help them with the intent of engaging in a therapeutic alliance with them. I then ask them something about themselves. Usually, I’m asking them about hobbies…getting to know a little bit about them works sometimes* (Paul, Doctor)
*I always enter my assessment with someone and say “hey I know that you had a really crap day today. I’m going to ask you some questions. If I’m starting to piss you off, can you let me know so that I can give you some space because I know that you don’t want to hurt or abuse me, and I don’t want it to happen either”…giving them that permission…[to] say stop* (Beth, Mental Health Clinician)
*I think there’s a real benefit in…clinicians and other treating professionals to actually just take a step back and listen to the patient…I think that’s the first step that we quite often forget to do…we forget to stop and actually just listen* (Charlie, Nurse)
*I find that…when you treat them like a peer, it’s often the first time they’ve been treated like that…it can really mend a lot of things, and get them on side* (Mark, Paramedic)
*I just try and get an understanding of what has caused them to be triggered in that instance…and then work alongside them…I really try and inform my patients that I’m here to advocate for you…we’re a team* (Mary, Paramedic)
*I think one thing that helps, and obviously you have to pick the right patient…but not standing over people. Your body language is so important and remaining calm…is the important thing…making sure you’re not crossing your arms…if it’s safe enough, kneel down next to them and not just lean over them* (Harriet, Nurse)
*Food would be our go-to with every kid…just things that you know are calming, hot blankets, those things are amazing* (Hazel, Nurse)
*It's about trying to be transparent…with the young person…I think that goes a long way, and I’m not sure people do that early enough. They can see…what’s happening…if you are picking it up already, then [the agitation] is probably already level 9 or 10…there’s a lot of power in just calling out what you are feeling…rather than dancing around it* (Judy, Mental Health Clinician)
*Using distraction, I think has been one of the better things. Whether they like games or books…or YouTube or whatever, there’s usually something* (John, Paramedic)
*Probably, oh, about 50% of the time I suspect we’re successful in just calming everything down without resorting to pharmacologic methods* (Julie, Doctor)
*You sometimes almost want to take that to the politicians and do-gooders like, “you should never sedate someone. You can always talk them down.” I’m like no you can’t. Anyone who’s actually worked with these patients says, “you can’t talk them down sometimes.”* (Mark, Paramedic)
Weighing up when to use medication and which route to choose
*Quantifying when there is in fact an urgent risk, is the most useful frame I find…it’s the minority of cases in kids who do need chemical sedation but when they do, it shouldn’t be deferred* (Joseph, Doctor)
*I’d certainly give them the opportunity to take something [oral] first. The phrasing is important. “Look, it’s just to take the edge off things, not to knock you out. It’s just to make you feel less agitated so we can have a proper talk in a little while.”* (Chris, Doctor)
*It's all to do with dangers…if everything’s okay, it’s basically verbal de-escalation…then as you go up [the level of agitation]…you’re going into oral [sedation]…and IM…it really depends upon the immediacy, the perceived dangers and everything going on at the time* (Peter, Paramedic)
*Some clinicians are keen that they’re absolutely only going to give oral medications to the point you’re chasing some kid around trying to hold them down…risk getting bitten, they’re spitting in your face. I think some cases, we need to have a bit lower threshold…to pull the trigger [for parenteral medication]* (Patti, Nurse)
Which medication to use
*I tend to stick to the same thing over and over* (Kate, Doctor)
*[Drug choice is] definitely variable depending on the child, the etiology, and circumstance. …there’s so many different encounters or flavours of encounters* (Joseph, Doctor)
*I have a lot more experience with ketamine…I try to do things that I know that I’m comfortable managing. I’ve given lots of kids ketamine. If I had an acutely disturbed kid…I’d be reasonably comfortable giving them an IM dose of ketamine* (Betty, Doctor)
Dosing
*My strategy is to give much more [medication] than I think I should give…now if this kid should get five milligrams, I say, “That’s fine. This kid is getting 10 to 15.”…that doesn’t always make everyone happy…but if I’m going to give an IM, I don’t want to…just make them angry….I’d rather give them one injection…I know the side effects and we’re in a place we can deal with all the side effects* (Harry, Doctor)
Medication in the prehospital setting
*We’re totally hamstrung by the fact that it’s now completely off the table to do anything without consulting the receiving hospital regardless of circumstance…I can think of a couple of cases where I’ve had an extremely agitated, let’s say 15-year-old who’s essentially the same size as me…and who’s violent, agitated and screaming…we get these recommendations for doses that we would deem to be inappropriate because it’s hard to paint the picture of how angry or violent a patient is when you’re not there* (John, Paramedic)

De-escalation strategies were discussed extensively. Verbal de-escalation was noted to be a nonrestrictive means of assisting the young person to achieve behavioral containment, but the participants highlighted a range of elements that required consideration for it to be successful. These included developing rapport with the young person while being transparent about your role and the boundaries of the therapeutic relationship. The participants found spending time listening to the young person’s story and providing acknowledgment and validation of their situation to be valuable. A range of nonverbal strategies were also employed, which included using an open posture and sitting down next to the young person whenever it was viewed as safe to do so. Participants also highlighted the benefits of offering food, drink, or other items of comfort to the young person.

Participants emphasized how the multidisciplinary approach assisted to not only help calm the young person but also support the team to work more effectively together. There were some instances where the participants felt that particular staff members had worsened the situation, but these appeared to be infrequent.

Despite best attempts, these de-escalation strategies did not always succeed. Participants spoke about the tension between wanting to use nonrestrictive measures but recognizing that these had failed, and needing to use more restrictive means, such as medications or restraint, to ensure the safety of the young person and staff. Participants agreed that the decision to provide medication needed to be carefully considered, but when it was necessary to ensure the patient’s safety, medication provision should not be delayed. There was considerable variability in the participant’s choices regarding the route of medication. Some advocated for concerted attempts to provide oral medications, even when the young person was profoundly escalated, whereas others used parenteral medication as the initial route of administration in these situations. The medication types provided also varied markedly. All participants agreed that if parenteral medication was being provided, a dose large enough to be guaranteed to achieve behavioral containment should be used.

##### 3.2.1.3 Risk of harm and personal impact

The challenges of balancing the patient’s safety with the safety of staff were a key consideration for participants (Table 4). The risk of physical violence faced when caring for these young people was emphasized, although many normalized this as being an accepted part of the job. There was a significant emotional impact on participants through providing care to these young people. This included feelings of exhaustion, hopelessness, helplessness, fear, frustration, and sadness. Participants also emphasized that caring for these young people can be emotionally draining.

**Table 4:** Occupational violence and aggression, emotional impact of care, and debriefing.

Risk of violence
*It was like…what is my threshold for feeling unsafe or being able to control the scene…but also balancing trying not to do future harm to [the patient]?* (Mary, Paramedic)
*You’re trying to keep that young person safe, you’re trying to keep yourself safe as well, and your colleagues* (Pamela, Paramedic)
Description of physical violence
*They are potentially high-risk people that can whip out a razor blade and stab you. We have to be careful with them. Our approach is probably being conservative to make sure that the staff are protected* (Kate, Doctor)
*They kicked off big time. They actually got a police gun out of a holster…fortunately the old copper was quite smart, there was nothing in it…it happened that fast* (Nathan, Paramedic)
*A kid at one stage threw a couch through the window* (Betty, Doctor)
*There’s been some pretty severe things that have happened here. Computers being torn off the desk and broken, a knife has been pulled recently* (Hazel, Nurse)
*He was wielding an axe in community…everyone was very afraid of him* (Nicole, Doctor)
*They will attempt to bite, and they just usually spit at you…this particular girl…she’s assaulted about 60 paramedics…I don’t think she actually ever gets in the ambulance [now] because she’s too violent* (Chloe, Paramedic)
Acceptance of physical violence
*I feel like you just get used to it, which is not the right way to look at it really…I feel like it’s almost a little bit acceptable that they’re like that even though it’s not okay to ever get hit* (Harriet, Nurse)
*We had a young person in, and it was a young nurse who went in to see this patient, and it was a mental health patient that came in quite escalated…the young nurse got kicked in the face, and the senior-level nurse said, “that serves you right for putting yourself in that situation”* (Patti, Nurse)
*As hard as it is, I guess letting them abuse [you], they’ve just got verbal diarrhoea at that time anyway. Often, I don’t think they’re really necessarily purposefully trying to call you those names, it’s just they’re angry. I guess just letting them let off some steam* (Hazel, Nurse)
Emotional impact of care
*These jobs potentially sit with me longer than other jobs, as in they’re the ones that I go home and think about. I think the reason, primarily, is because there’s a sense of helplessness. I feel like we’re probably just one cog that contributes to this ongoing wheel for this young person…the service system…it’s falling short in meeting that young person’s needs…there’s this sense that…I’m contributing to this really traumatic journey in and out of health services* (Pamela, Paramedic)
*Sometimes I would be absolutely petrified, very fearful that I was going to be assaulted* (Anthony, Mental Health Clinician)
*It’s disturbing at times how they do present and how they speak to you and some of the threats they might make. I don’t believe a lot of the threats, but you have to take them seriously…the most recent one where I did have the threat of, “I’m going to murder you*—*I’m going to kill you when I get out of this room’…this was a night duty…it’s so challenging to go home…go to bed with that in your brain* (Hazel, Nurse)
*It depends on my own bias I bring at the time. If I look at this patient and think, ”you’re doing this on purpose“…I feel frustrated…for people that I think that they don’t really have control…over the way they’re behaving…I feel a degree of responsibility…and a degree of empathy…and a degree of sadness that whatever’s happened that has led them to this circumstance* (Harry, Doctor)
Debriefing
*We don’t really talk about [acute behavioral disturbance] a lot. We’re always talking about the…cardiac arrest, and the big traumas…but not the mental health…I’ve touched base with [the paramedics involved]…and they’ve actually reached out…and said, ”I can’t believe what we did the other night.“ It obviously impacts them* (Nathan, Paramedic)
*I really do think that we would benefit from debriefing in those situations, particularly when we’ve had to, say, restrain a 16-year-old…we never want to restrain people…I think a debrief would really, really benefit us in improving our management* (Charlie, Nurse)

Participants believed that there were inadequate opportunities to debrief after caring for young people with acute behavioral disturbance. This was highlighted as being in contrast to medical events of similar severity, which were commonly followed by a debrief due to the acuity and stress of the situation. Participants questioned why these high-acuity psychiatric events were not approached in a similar manner.

##### 3.2.1.4 Workflow, resource, and bystander impacts

The challenges of managing these young people in an underresourced health system were discussed (Table 5). Participants worried about the effect that these presentations had on other patients in the ED, in particular whether other patients or families would feel frightened after witnessing the young person with behavioral disturbance being aggressive or violent.

**Table 5:** Resource limitations, disposition, and follow up.

Resource limitations
*I had a few instances where we had transported [a patient with behavioral disturbance], and then we were just ramped∗….as you know, sedation doesn’t last for ages. Then you’ve got a patient that’s becoming heightened in an ED…they don’t have any beds…you’ve got other paramedics looking to you and sometimes being like, what are you doing to control your patient?…These can be very long jobs when you’re met with that ramping…it’s not the hospital’s fault. It’s the state of healthcare. It does make it hard* (Mary, Paramedic) ∗“Ramping” refers to an off-stretcher delay where paramedics are unable to complete the transfer of clinical care to the ED within an appropriate timeframe due to a lack of staff or space within the ED, usually as a result of access block.
Effect on other patients
*If I go in to get involved in [the patient presenting with acute severe behavioral disturbance], then I’m going to neglect the rest of the emergency department because that’s usually what that presentation needs to get the endpoint for that patient that they need. I might sit there for an hour, if required, and just let the place go to shit* (Sam, Doctor)
*A thing we worry a lot about here is…we have a situation where someone escalates and they’re running around and aggressive and maybe verbally aggressive and violent, they’re in really close proximity to other kids, other children, and so you always are a bit scared about the repercussions of that and what could potentially happen* (Annabelle, Nurse)
Disposition
*The negotiation about where next is also a very tricky one, because if you have someone who is agitated, the wards…are not really well set up for someone who is likely to become aggressive and agitated…often, they need to go to the psychiatry ward, even though they’re not really a psychiatric patient, just because the behavioral management needs to be done…sometimes you get children…particularly the autistic spectrum ones that are not safe to go to the ward, who don’t need to go to psychiatry…they’ve spent several days in the emergency department trying to find an appropriate place…it does get very complex* (Chris, Doctor)
Community support and follow-up
*There’s no quick fix…but you know that person’s going to need so much more when they leave here for proper therapy…does it ever get done? I don’t know. Sometimes we see them again and similar situations happen* (Hazel, Nurse)
*I think the thing with me that I kind of – I think…all the support places, they say, “oh, yes, and if this happens phone 000 and go to the ED.” People just view us as, go to the ED and it’ll fix it, and I get it. We’re open 24/7, there’s always people here. You can be expected to be safe here, but also it’s like there’s still nothing we can do* (Cassandra, Mental Health Clinician)
*From our perspective…it’s that pattern. This is what we do, we go, we treat, we transfer the young person back into the community. We go, we treat, we transfer…despite all attempts to try and coordinate…we don’t seem to come up with effective ways to support that young person to not have to navigate that cycle* (Pamela, Paramedic)

Problems with timely and appropriate disposition for this group of patients were also emphasized. This was discussed by paramedic staff who spoke about the challenges of keeping the young person calm while they remained on an ambulance trolley in the ED corridor due to a lack of available ED beds. Often, the paramedics were caring for these young people many hours after they arrived at the ED. Medical staff detailed the challenges of disposition for these young people, in particular determining the appropriate inpatient specialty to provide them with ongoing care. Many felt that the inpatient wards were not adequately resourced to provide care to young people with behavioral disturbances.

## 4 Limitations

Participants included in this study may have self-selected to participate because they had an interest in the management of young people with acute severe behavioral disturbance or because they had cared for a challenging young person. As a result, the findings may not represent the experiences of all health care professionals looking after these young people in the acute care setting. Participants for our study were recruited from the Australian context, which may limit the generalizability of the findings.

Because of governance and logistical challenges, we were not able to interview police or security staff who are the other key staff groups involved in the care of these young people. They would be an important group to be included in future work.

## 5 Discussion

To our knowledge, this is the first study to assess the experiences of health care professionals looking after young people with acute severe behavioral disturbance across the prehospital and ED environments. Common themes emerging from the interviews across craft groups included contributing etiologic factors, the management approach, the physical and emotional impacts of this patient cohort, and the implications that they have on staff workflow within the acute care setting.

Participants identified that young people with mental health conditions and neurodevelopmental disorders were, in their experience, at high risk of developing acute severe behavioral disturbance. This is consistent with existing literature which supports a mental health complaint as being the most commonly associated etiologic factor.^3^^,^^16^ Young people with neurodevelopmental disorders—in particular autism spectrum disorder—are overrepresented in the cohort of young people presenting with acute behavioral disturbance.^17^^,^^18^

Participants discussed in detail their approach to the management of these young people. This included a concerted focus on a range of de-escalation strategies. Although these strategies are widely advocated in both the literature^19^ and existing behavioral disturbance management guidelines,^20^ the evidence base for the effectiveness of any of these strategies is limited.^21^ Participant approaches to medication use were highly varied when they were considering when to provide medication, what route to use, and which medication to provide. This is not entirely surprising because currently there is no prospective observational or randomized controlled trial-level evidence to guide which are the most effective medications in this patient cohort.^22^ Further research is urgently needed to compare commonly used nonpharmacologic and pharmacologic strategies to determine which are the most effective in achieving behavioral containment in the acute care setting.

The responses from participants in this study reinforce the previously reported high rates of occupational violence within the prehospital and ED environments.^23^, ^24^, ^25^ Because of the regularity with which it occurred, participants appeared to accept this as being a normal part of working within the acute care setting, a phenomenon that has previously been highlighted by nursing staff in the ED.^26^

Participants in our study spoke about the considerable emotional impact of caring for young people with acute behavioral disturbance across the acute care sector. Previous qualitative work has emphasized the moral distress experienced by staff caring for young people with mental health conditions.^27^^,^^28^ The onflow effects of moral distress are considerable and include staff attrition and the increased risk of patient safety incidents.^29^

All craft groups participating in this study outlined the perceived benefits of clinical debriefing. Previous literature highlights the positive benefits of debriefing after the management of a pediatric behavioral disturbance event, specifically in relation to improved team cooperation and communication and by allowing participants to process a range of emotional reactions.^30^ Despite these positive consequences, clinical debriefing for mental health may not be standard practice in the acute care setting, with research in the adult behavioral disturbance cohort instead highlighting a culture of staff coping with these potentially traumatic events on their own.^31^ Determining the utility of debriefing in this clinical scenario with the aim to drive policy and practice change is a research priority.

In conclusion, health care professionals working in the acute care setting highlighted a number of common experiences when caring for young people with acute severe behavioral disturbance. Participants found caring for these young people emotionally challenging and experienced or witnessed occupational violence frequently when managing this cohort. Front-line clinicians aim to provide care to these young people using the least restrictive means feasible while ensuring patient and staff safety. Currently, there is limited high-quality evidence to guide clinicians as to the effectiveness of any of these strategies, highlighting a key area for future research.

## Author Contributions

E.M.B. conceived the study with input from F.E.B., J.K., and S.S.C. E.M.B. obtained ethics approval for the study and Z.N. obtained governance approval from Ambulance Victoria. E.M.B. obtained the data. E.M.B. and N.D. analyzed and interpreted the data. E.M.B. drafted the initial manuscript, with all authors contributing substantially to its revision. E.M.B. takes responsibility for the paper as a whole.

## Funding and Support

The study was supported in part by a grant from the Medical Research Futures Fund (NHMRC ID: 1179137), Canberra, Australia, and supported by the Victorian Government’s Infrastructure Support Program. E.M.B. is supported by an NHMRC postgraduate scholarship (ID: 2005279). Z.N. is supported by a National Heart Foundation fellowship. F.E.B. is supported by an NHMRC Investigator Grant (ID: 2017605) and the 10.13039/100014607Royal Children's Hospital Foundation, Parkville, Australia.

## Conflict of Interest

All authors have affirmed they have no conflicts of interest to declare.
