# Supplementary material for: Qualitative Exploration of Health Care Professionals’ Experiences Caring for Young People With Acute Severe Behavioral Disturbance in the Acute Care Setting
Source: J Am Coll Emerg Physicians Open. 2025 Jan 13;6(1):100030. doi: 10.1016/j.acepjo.2024.100030 (PMC11852700; doi:10.1016/j.acepjo.2024.100030)
Supplement: Appendix A [file mmc1.docx]

**APPENDIX A**

**Interview Guide**

Interviews will be conducted using broad topics to guide discussion between the researcher and the participant. Examples of questions relating to each topic are provided along with examples of prompts used. Because it is anticipated that the interviews will follow a conversational style and will be guided by the participant’s responses, topics will not always be covered in a particular order nor will questions be asked using the exact wording outlined below.

Introduction from the researcher

“Thank you for taking the time to participate in this interview. My name is <researcher’s name>. I am a researcher at the Murdoch Children’s Research Institute (MCRI).

Today I will be interviewing you about your experiences of managing children with acute severe behavioural disturbance (ASBD) in the Emergency Department/Pre-hospital setting. The interview will be fairly informal, more like a conversation. I will ask you questions about a number of topics including your experiences treating children with ASBD, your strategies in managing them, which medications you preferentially use (if applicable) and how you decide which medications you use. I am conducting this interview purely for research. If there are any topics that you do not feel comfortable discussing, please let me know and we will move on to the next topic.”

| **BROAD TOPIC** | **EXAMPLE QUESTION** |
| --- | --- |
| *General conversation starter* | Can you tell me a bit about your experiences caring for children with acute severe behavioural disturbance? |
| *Management of ASBD* | What is your general approach to management of a child with ASBD in (your setting)?  Have you found any strategies that are particularly effective in managing these children?  How would you describe or define de-escalation in this setting if you use this technique?  Can you tell me a bit more about your experience in using de-escalation to manage these children?  How do you feel managing these children?  Have you experienced any particular emotional response?  Do you have any particular concerns around managing these children?  For clinicians:  Do you have set medications that you tend to use when managing paediatric ASBD or do these vary depending on the situation? (If relevant to the participant) |
| *Extra information* | Is there anything else that you would like to talk about in relation to paediatric ASBD today?  Is there anything you feel like we haven’t discussed that you would like to add?  How did you find the interview overall?  Is there anything that you think I could change for the future with my interview technique/style? |
