# Supplementary material for: Qualitative Exploration of Health Care Professionals’ Experiences Caring for Young People With Acute Severe Behavioral Disturbance in the Acute Care Setting
Source: J Am Coll Emerg Physicians Open. 2025 Jan 13;6(1):100030. doi: 10.1016/j.acepjo.2024.100030 (PMC11852700; doi:10.1016/j.acepjo.2024.100030)
Supplement: Appendix B [file mmc2.docx]

| Appendix B | | |
| --- | --- | --- |
| Thematic codebook used for the qualitative analysis of health care professional experiences managing young people with acute behavioral disturbance. | | |
| Code | Subcode | Example Quotes from Participant Transcripts |
| 1. Contributing factors | |  |
| 1.1. Potential triggers | Mental health | *They were just feral…they just behaved like a caged rat…they’ve got severe complex trauma background and attachment disorders…[they] said ‘Oh yes, I’m going to kill myself’* (Betty, Doctor)  *The most recent child…came in as a 17-year-old who is in that horrible prodrome of schizophreniform illness…his violence, which is dissociative, it’s almost like a guarded dissociative state where he seemed to randomly just go completely insane and want to hurt anyone that was near him and smash everything* (Sam, Doctor)  *I think what we see in terms of acute behavioral disturbance is probably two, maybe three groups. There’s certainly a smaller section that are perhaps a true psychosis…probably the areas that we see it more…where they’re emotionally dysregulated in relation to their suicidal ideation or not having their needs meet* (Cassandra, Mental Health Clinician) |
|  | Neurodevelopmental disorder | *The kids with autism…or other intellectual disability who aggression is their way of expressing frustration…I think the worst…behavioral patients I’ve looked after fit into this category…[the parents] get to the point where they just say ‘my kid’s now 15 and he’s too big, I can’t restrain him…he’s breaking all my walls* (Harry, Doctor)  *The other cohort is the autistic teenagers that weight 120 kilos that are bashing their head against the glass…and bleeding everywhere…these people are coming in with say six police officers and ambulance staff* (Kate, Doctor)  *When they are aggressive, they’re not easy to control, and they tend to destruct everything. If they start assaulting Mom or Dad, we end up there…it is frightening to see when there’s a six-foot man who’s actually 15 smashing a house and like ‘Oh, this is terrifying’* (Chloe, Paramedic) |
|  | Substance or admission seeking | *We’re creating a reward-type cycle, where it’s okay if I do this, ambulance comes, I get this, I go there, but do you know the reward potentially is the medication?* (Pamela, Paramedic)  *I’ve dealt with a couple of children that have been, I think, rather obviously seeking a certain pharmacology…it’s a really challenging situation to be in because you have this kid screaming at you, ‘just give me the ketamine’…I find it a really difficult situation to navigate* (John, Paramedic)  *The difficulty is with our mental health system, it’s designed in a way where – it sounds terrible, but the squeaky wheel gets the oil. If you are in an emergency department and you keep calm, you’re collected…more likely than not, you’ll be discharged…whereas if you go to the ED and say ‘I’m going to effing punch you in the face. I’m going to do this, I’m going to do that, if you let me go’ then I would say 95% of the time that young person will be admitted. I think there’s…a cultural dynamic where they learn that is the case. That exacerbates behaviors of concern when hitting the ED* (Anthony, Mental Health Clinician) |
|  | Environmental factors | *One thing that I notice escalates…kids…are long waits….it’s the nature of the emergency department, but it’s hard to watch someone escalate purely just because they’re frustrated on waiting* (Harriet, Nurse)  *Acute behavioral disturbance can be the screaming child because they cut their finger on glass and they’re frightened of blood* (Paul, Doctor)  *We got them in the back [of the ambulance] and we can control the environment, we can make it calm…we would ask her what music…she wanted to listen to…we can turn the lights off…we don’t need to be doing a blood pressure very five minutes* (Mary, Paramedic) |
|  | Organic illness | *I walked past [pediatric bay] and witnessed a commotion of four or five people trying to sedate a young girl of about 11 years old…they were trying to hold her down…the short version for that story is that she was severely hypoxic…I just wanted to flag…to think hypoxia, hypoglycaemia, all the organic causes* (Arthur, Doctor) |
| 1.2 Parental conflict or maladaptive parenting styles |  | *The couple of times that [involving the parents] hasn’t worked, it’s been because there has been a conflict with the parent. The conflict between the child and parent has been the reason why everyone’s escalated* (John, Paramedic)  *I’ll be honest…a lot of the times we are called for pediatric behavioural disturbance…it’s often…poor parenting…some people are just good at certain things and some are just bad…as a result, you are going to get kids whose parents are just, ‘we’re going to call an ambulance for them. We can’t handle this’. You’re like ‘what do you mean? They’re a salty teenager. They’re a confused 12-year-old going through puberty and they’re lashing out. You’ve got to deal with this’* (Mark, Paramedic)  *If the parent’s dysregulated, that actually might be the trigger that set the kid off…often the parents coming or the carers…they’re not in the best of places* (Angela, Mental Health Clinician)  *The other thing is their upbringing too. A lot of these young people…have traumatic upbringings, parenting styles that are quite neglectful or abusive. They have these role models so the expectation that we’re going to change that behaviour overnight, it’s just unreasonable* (Anthony, Mental Health Clinician) |
| 2. Management strategies | | |
| 2.1 General approach |  | *We’re all humans, we’ve all got human rights. It’s trying to do the least restrictive mode…that would help them. Whatever we do…it’s for their benefit. It’s not you’re shitting me, so therefore, I’m going to jab you or tie you up. It’s like, ‘I think your brain and your body needs a rest and we need to sort that out and we cannot either rest it by you calming down, by me chatting to you, so we can rest it by me giving you something that will make you calm’* (Ben, Doctor)  *If there’s a situation which you need to get control of, you’ve got your…verbal or distraction de-escalation, empathizing with their situation, offering to fulfil their needs…really then in kids trying to avoid sedation or chemical restraint or even any or even any sort of invasive restraints which hinge upon their autonomy* (Joseph, Doctor)  *It's all to do with dangers…if everything’s okay, it’s basically verbal de-escalation…then as you go up [the level of agitation]…you’re going into oral [sedation]…and IM…it really depends upon the immediacy, the perceived dangers and everything going on at the time* (Peter, Paramedic) |
| 2.2 De-escalation | | |
| 2.2.1 Verbal de-escalation | Develop rapport | *You need to know how to be able to talk to them. Doctors who are very doctor-like and very prim and proper and talk the medical language, they’re not going to get through to a 15-year-old that’s going off* (Ben, Doctor)  *Having the single voice…and feeling responsible and able to provide that communication, it is best to have that single person being clear and concise* (Joseph, Doctor)  *I tend to be pretty relaxed…sit down and I usually just have a social chat if I can first to get a feel for what’s going on…try and make them as comfortable as I can* (Sally, Doctor)  *I sit on the end of the bed. I introduce myself. I tell them I’m here to help them with the intent of engaging in a therapeutic alliance with them. I then ask them something about themselves. Usually, I’m asking them about hobbies…getting to know a little bit about them works sometimes* (Paul, Doctor)  *With [frequent attender] they talk about animals and horses and pictures of dogs and things like that…utilizing things like that to assist you with the de-escalation process* (Peter, Paramedic) |
|  | Boundary setting | *There is real importance particularly in children and adolescents to say what your boundaries are, and then make sure they understand that is a boundary. You can negotiate and give people options, but they’re very limited options, ad making sure when they do try and cross the boundary…then you have to say ‘No, you’ve crossed the boundary, we’re no longer negotiating’* (Chris, Doctor)  *It’s really…giving them choices around boundaries for their behaviour…if we can have this discussion maybe we can move forward and get a plan that’s safe for you* (Nicole, Doctor)  *There needs to be consequences for behaviour…they’ve been behaviorally ok…they’ve calmed down…let’s move [out of the seclusion room] rather than just hanging around”* (Kim, Mental Health Clinician) |
|  | Verbalising safety | *I just say like ‘I need to feel safe, and everyone in this ambulance needs to feel safe. Do you feel like you are going to engage in behaviour that might…lash out, are you going to spit?’…and give them an opportunity to be like ‘yes, I am feeling like I might escalate’, and check in with themselves* (Mary, Paramedic)  *I always enter my assessment with someone and say ‘hey I know that you had a really crap day today. I’m going to ask you some questions. If I’m starting to piss you off, can you let me know so that I can give you some space because I know that you don’t want to hurt or abuse me, and I don’t want it to happen either’…giving them that permission…[to] say stop* (Beth, Mental Health Clinician)  *The kids that use violence, as well as threats of violence…I emphasize that they deserve to be safe, we deserve to be safe…sometimes I’d just say the word ‘safe’ 10 times to the point where they go ‘all right, you said it’* (Carmel, Mental Health Clinician) |
|  | Being transparent | *Sometimes, we can go in with really good intentions and be like ‘Don’t do this, don’t do that’ but we’re not actually giving them permission to say that they’re feeling out of control or that they’re starting to unravel…providing that feedback to them saying ‘When you’re getting up and walking around, it makes me feel like you’re a bit annoyed…how would I know that you’re starting to escalate?’* (Beth, Mental Health Clinician)  *Give them choices…’I find it hard to talk to you when you’re pacing. Do you need a bit of time or do you want me to come back later?’* (Nicole, Doctor)  *It's about trying to be transparent…with the young person…I think that goes along way, and I’m not sure people do that early enough. They can see…what’s happening…if you are picking it up already, then [the agitation] is probably already level 9 or 10…there’s a lot of power in just calling out what you are feeling…rather than dancing around it* (Judy, Mental Health Clinician) |
| 2.2.2 Listening to their story |  | *I just think that it’s about empathy...I had a young [First Nations] boy crying, and I felt really bad. His girlfriend said ‘Oh, no, he’s crying because you’re the first blokes who’s had a proper listen’* (Arthur, Doctor)  *I think early intervention with anybody who can be a well-trained active listener. We use a lot of social work, mental health team…ancillary staff that don’t necessarily need to be nurses or doctors…which seems to help greatly* (Patti, Nurse)  *A lot of doctors feel that these patients are wasting my time…that mentality and therefore they don’t take the time…take half an hour, it’s okay. Half an hour, if you take half an hour, then you don’t have to inject someone…just by talking* (Ben, Doctor)  *I think there’s a real benefit in…clinicians and other treating professionals to actually just take a step back and listen to the patient…I think that’s the first step that we quite often forget to do …we forget to stop and actually just listen* (Charlie, Nurse)  *It’s really about that acknowledgement, that validation, that whole person-centred care approach that ‘I’m here’* (Kim, Mental Health Clinician) |
| 2.2.3 Treating them with respect |  | *I want to give them that 100% and then they feel valued. A lot of the time, they don’t feel valued. They just feel as though ‘oh here we go again’* (Nathan, Paramedic)  *I find that…when you treat them like a peer, it’s often the first time they’ve been treated like that…it can really mend a lot of things, and get them on side* (Mark, Paramedic)  *I think part of it is…I become the first adult, that’s actually spoken to them, quietly and respectfully, during the last…how long their episodes been going on…it’s a low-hanging fruit to go in and speak respectfully…it puts you on their radar as someone that’s not a threat* (Sam, Doctor) |
| 2.2.4 Non-verbal strategies | Body language | *I think one thing that helps, and obviously you have to pick the right patient…but not standing over people. Your body language is so important and remaining calm…is the important thing…making sure you’re not crossing your arms…if it’s safe enough kneel down next to them and not just lean over them* (Harriet, Nurse)  *I think if you walk in with a non-judgemental approach and without compassion fatigue that is very evident to the people in the room what your approach will be from your body language and your demeanor. That’s really, really, really you just absolutely have to get off on the right foot* (Arthur, Doctor)  *Sometimes, as much as I shouldn’t, but sometimes I’ll sit down next to them…if they’re sitting on the floor, I’ll go sit on the floor with them. People look at me strange sometimes, but it’s those little things that you do that really do break the barriers* (Nathan, Paramedic)  *I’ll sit down, be below them, be small…try and encourage their cooperation* (Julie, Doctor) |
|  | Maintaining a sense of calm | *I think what really helps with those kids is, really to not take it personally…they say some pretty horrendous things…attempting to spit on you, and all that…I think if you can…maintain that calm…was usually enough to have them come down* (Carmel, Mental Health)  *Even though they’re abusive. Just trying to let that just go over your head, not be emotional…even though that’s the first thing you want to say when they’re calling you all the lovely names that they sometimes call you but just letting that completely go over your head and being not emotional* (Hazel, Nurse) |
|  | Food, drink, items of comfort | *Simple techniques like offering them a cup of tea and a sandwich and a warm blanket, and trying to empathize [with them]* (Ben, Doctor)  *Food would be our go-to with every kid…just things that you know are calming, hot blankets, those things are amazing* (Hazel, Nurse)  *Kids love food…it’s a really good form of engagement…I gave her some popping candy…and we focussed on what was going on with her in her mouth…she calmed down. It was phenomenal* (Beth, Mental Health Clinician) |
|  | Low stimulation | *For these highly disturbed kids on the autism spectrum, actually shutting them up in a room…it’s much quieter…there are no things that go ping…a low stimulus environment* (Betty, Doctor)  *Having a behavioral assessment room…where they can get away from all the noises is helpful…they just need somewhere they are away from those…sensory overloads – the noises, the action, the people looking at them* (Chris, Doctor)  *We can have the room dim and…to be soundproof to the rest of the noise of the ED* (Kate, Doctor)  *It’s really important not to overstimulate…quiet room, softly spoken…so that you are not bombarding that child…stepping back more than overbearing* (Annabelle, Nurse) |
|  | Bystanders worsening the situation | *That includes…being cognizant of a milieu of stimuli…potentially family members, friends, or even staff members might be potentially exacerbating the behaviours that we’re trying to respond to, and trying to mitigate that by potentially having those individuals separated from the young person…to support them to get to a space where they’re better able to regulate* (Anthony, Mental Health Clinician)  *The way security responded were really in your face…it just escalated and escalated to the point that this kid spat blood in the security guard’s face and then he was dragged outside…as soon as I got him into a bay and closed the curtain…and it was just him and I, he just calmed down…the escalation could have completely been avoided if staff had just stopped and listened* (Charlie, Nurse)  *Remove the case of thousands, because that can be very, very overwhelming* (Chloe, Paramedic)  *I brought the Mom upstairs and I was like, maybe you can just talk to her and help to deescalate her. It sounded like at the time that was a good decision because she had a good relationship with her mother…it did not go to plan. She started yelling at screaming at the Mom, referring back to her trauma, saying, you know what happened to me…and you turned a blind eye. You were meant to protect me* (Mary, Paramedic) |
|  | Bystanders assisting the situation | *Allowing the boyfriend to be there if that’s what really keeps a lid on someone, why would you not want that…why wouldn’t you want to just keep that child calm in any way or form* (Hazel, Nurse)  *The family is far and away the best resource - they always seem to have a really great understanding of how the kid’s going to react to different things and what works and what doesn’t* (John, Paramedic) |
|  | Distraction | *Using distraction, I think has been one of the better things. Whether they like games or books…or YouTube or whatever, there’s usually something* (John, Paramedic)  *I’d rather leave them in the courtyard…put them out there, give them some sporty equipment, kick a ball around. Give them a chance to calm down and the parents a change to calm down…as well* (Cassandra, Mental Health Clinician) |
| 2.2.5 Pre-existing relationship between the young person and staff |  | *I find most of the time, actually, there might be a staff member that knows that patient well and is willing to become involved in the behavioral crisis…one particular nurse might’ve looked after them…and has rapport* (Joseph, Doctor)  *There may be…members on scene that know this young person that has attended before and…already [has] an established relationship…there’s a strong need to have someone there that knows that young person so that then we can start looking at some strategies to support and de-escalate* (Pamela, Paramedic)  *Knowing a little bit about the kid…a lot of the kids at the moment that we’re seeing are people that we see often…familiar people is really helpful for them because that in itself can be a bit containing* (Beth, Mental Health Clinician)  *I do remember each one I’ve assessed…it helps them not having to repeat the story* (Kim, Mental Health Clinician) |
| 2.2.6 Providing the young person with control over their care |  | *I just try and get an understanding of what has caused them to be triggered in that instance…and then work alongside them…I really try and inform my patients that I’m here to advocate for you…we’re a team* (Mary, Paramedic)  *I generally like to give them as much agency as I can…just little bits of choice where you can give it to them* (Mark, Paramedic)  *I always want them to have that empowerment. I want them to feel like they have an element of control of what goes on* (Kim, Mental Health Clinician) |
| 2.2.7 Role of members of the team | 2.2.7.1 Teamwork | *We have excellent support from security...just letting the kids insults slide of them…and making themselves not a target for the kids…we have a pretty good working relationship…we have an exchange about what their assessment is, what my assessment is…you have to…trust what they’re doing and listen to each other* (Betty, Doctor)  *It's more like involving other people, getting the mental health [clinician]…to be there and they often have a really good approach as well. It’s different to mine and sometimes I’ve antagonized people, and the mental health people don’t antagonise people…by using that team-based approach of either yourself and nurse or yourself and mental health [clinician] or a combination of those three…you’re getting a good result* (Paul, Doctor)  *I think [the ED] is such an incredibly supportive environment…everyone checks in with each other, and I think that goes a long way as well to manage these more difficult patients. It doesn’t feel like you’re ever going to be criticized for actually going, I’m not sure how to approach this, or I’m feeling uncomfortable* (Judy, Mental Health Clinician)  *I have to say, generally we’re very, very lucky…all of our consultants are really good. If we say ‘hey, no. this isn’t going to work.’ They’re like ‘okay cool. What do you want to do…what are you comfortable doing?’ We’re very fortunate.* (Patti, Nurse) |
|  | 2.2.7.2 Police and paramedic relationship | *I think the police do a really good job…when there has been pressure [from police]…they’ve been on scene for a while, and they’re physically becoming exhausted from holding, restraining a patient…when they are getting to that threshold…you do feel pressure…definitely have felt pressured in the past by them to make decisions* (Mary, Paramedic)  *If [the setting] is, for example…a train station or train tracks or a very public place on a major highway, we really need the police to take the lead around the safety component. That’s generally what happens…the multi-agency response is really common* (Pamela, Paramedic)  *The cops are really, really good at establishing a rapport, particularly if they’re the first on scene. If that’s how it works out, I like to maintain that…if they already have a rapport and they’ve already relaxed a little bit…may as well keep that going* (Chloe, Paramedic)  *We’re very hands off as paramedics now…we’ve very much let the police do the physical restraining…we’re not trained…in terms of holding people down…you can do real harm if you’re not trained properly* (John, Paramedic) |
|  | 2.2.7.3 Role of mental health clinician | *We’re very fortunate to have the [mental health] team that we have…who will come and assess our kids…24/7…once they’ve seen the patients, made an assessment, then they will do things like follow up phone calls, liaise with the community mental health services, and so on…they’re good at de-escalating them because they actually know them, and also because they’re practiced at it* (Betty, Doctor)  *I was involved in the care of these kids…everything from them arriving actually at the emergency department, and in the ambulance bay…to managing to conduct…mental health assessment, including a risk assessment…and then doing planning around where to next…I have the view that any acute behavioral disturbance…fell within the remit of the mental health team to some degree* (Carmel, Mental Health Clinician)  *I can’t…write a script to give them risperidone or something…its actually little that can be done from that end for me. I often think a large part of my role in the moment on the shift is to make sure the parents and the carers have someone to listen to them. I think that is often one of the most containing things that you can do, take the time to hear them out…give them some supportive counselling* (Cassandra, Mental Health Clinician) |
|  | 2.2.7.3 Role of security staff | *Security…it’s good sometimes to have them on the off chance that we need them…[but] in this scenario security were really intense…right in this face, and he was taking a step back and then they would move forward. I’m like ‘hang on, guys. He’s actually trying to get away from you. Can you stop getting in his face?’ That’s why the situation progressed to where it did* (Charlie, Nurse)  *We’re always talking about security, I feel when I reflect on it, that’s probably been a really big predictor of where it goes depending on the security guard and their approach* (Judy, Mental Health Clinician)  *I think that the decision to grab a child, hold them down…it’s a big thing…security won’t really take that step into their own hands* (Chris, Doctor) |
|  | 2.2.7.4 Seniority of involved medical staff | *It has to be a senior-led service. It just absolutely has to be…I think it’s an art form* (Arthur, Doctor)  *They’re a special group…the acute behavioral disturbance [children]…it’s important to have some senior leadership…when it comes to an agitated kid, you might just need to…[think] outside the box…you need a senior registrar or consultant…because otherwise, it’s a fucking disaster* (Paul, Doctor) |
| 2.2.8 Need for early recognition and escalation |  | *What I’ve noticed is people aren’t identifying things early enough around dysregulation and arousal…you are stuck trying to develop a rapport or trying to figure out what to do when the crisis has already hit…everything we do is around parameters and criteria…how can we utilize what we’re already doing for physical health for someone’s arousal level?* (Beth, Mental Health Clinician)  *There still seems to be a culture when I’ve talked to nurses…feeling like they’re going to get some criticism…for calling a [emergency response team]…which again, I think feeds back into…letting things go too long…when they’re seeing that young person getting heightened* (Judy, Mental Health Clinician) |
| 2.3 Failed de-escalation |  | *I think as paramedics sometimes we’re not left with a lot of options…there’s some de-escalation strategies and steps…and then we are looking at chemical restraint…that’s something we don’t want to do…I think most paramedics will take the time and then try everything that they possibly can to engage and build rapport* (Pamela, Paramedic)  *You sometimes almost want to take that to the politicians and do-gooders like, ‘you should never sedate someone. You can always talk them down.’ I’m like ‘no you can’t. Anyone who’s actually worked with these patients says ‘you can’t talk them down sometimes.’* (Mark, Paramedic)  *Probably, oh, about 50% of the time I suspect we’re successful in just calming everything down without resorting to pharmacological methods* (Julie, Doctor) |
| 2.4 Medication | Decision to provide | *Quantifying when there is in fact an urgent risk, is the most useful frame I find…it’s the minority of cases in kids who do need chemical sedation but when they do, it shouldn’t be deferred* (Joseph, Doctor)  *I always think about it when I’m sedating someone…my thought is, okay, I’m going to put you to sleep, and I hope when you wake up, you’re feeling better…the reason for putting someone to sleep is to sedate them to a state where everyone is calm and relaxed, is to give them a chance just for their brain to settle down* (Chris, Doctor)  *[When giving parenteral sedation] you’re going to deprive them of their right to consciousness, effectively…for their own safety…that’s a really big deal, to literally go to walk up to someone, jab them with a needle, make them pass out, strap them to a bed and drive them to hospital…that’s how you’ve got to think about it* (Mark, Paramedic)  *I’m definitely someone that if I know the kid’s got a history of aggression and I can get oral medication into them, I will…the ED [environment] is tricky because the way the child’s programmed and the way we look at arousal, our environment’s not set up to support that* (Beth, Mental Health Clinician) |
|  | Timing | *I’m always proactive for some orals earlier if we can and would say to them, ‘is there any way we could give you some sedation maybe because I can see you are really distressed. I think it’s just going to take the edge off because you’re probably going to be waiting a little bit’* (Hazel, Nurse)  *Sometimes I find…we leave it too long…I know we’re quite big on de-escalating before going to things like IM…but I just find that letting someone go and hitting their head against a wall for a period of time, I don’t know…* (Harriet, Nurse)  *I do think that one of the first things that we do jump to is to provide them with medication…I think sometimes it is just necessary…it’s like, yes, that kid probably will calm down within an hour on their own…but at what cost to the broader emergency department and at what level of disruption? I think it is sometimes a little bit of a trade-off* (Cassandra, Mental Health Clinician) |
|  | Route of administration | *Verbal de-escalation, yes, it’s in textbooks. If they’re truly that behaviorally agitated that they’ve required many police and ambulance [staff] to bring them in and they’ve already had…on route IM and they’re still going off…then I don’t even bother with verbal de-escalation. Generally, they will refuse orals…I tend to just go straight to IM medication* (Kate, Doctor)  *I’d certainly give them the opportunity to take something [oral] first. The phrasing is important. ‘Look, it’s just to take the edge off things, not to knock you out. It’s just to make you feel less agitated so we can have a proper talk in a little while.’* (Chris, Doctor)  *The debate…is IV versus IM. Personally, I am an IM guy…the reason is, is because it’s more predictable in sedating people…[and] I’m not in the business of trying to get a drip in anyone who’s going berserk. It’s just too risky, but for the benefit, it’s not that great* (Ben, Doctor)  *I’m not a big fan of IM sedation. I’m a bit of a control freak. I do worry about sometimes just with your resources and so on that I prefer to have a little bit tighter control, just getting some IV sedation, so we can get things happening quickly and minimize the time that we’re doing physical restraint…but I will use IM sedation if I have to, just to be able to get at them* (Sally, Doctor)  *I think if they’re really escalated and [the doctors] try and give orals…you just know…it’s not going to work* (Harriet, Nurse)  *Some clinicians are keen that they’re absolutely only going to give oral medications to the point you’re chasing some kid around trying to hold them down…risk getting bit, they’re spitting in your face. I think some cases, we need to have a bit lower threshold…to pull the trigger [for parenteral medication]* (Patti, Nurse) |
|  | Choice of medication to provide | *I have a lot more experience with ketamine…I try to do things that I know that I’m comfortable managing. I’ve given lots of kids ketamine. If I had an acutely disturbed kid…I’d be reasonably comfortable giving them an IM dose of ketamine* (Betty, Doctor)  *[Drug choice is] definitely variable depending on the child, the aetiology, and circumstance. …there’s so many different encounters or flavours of encounters* (Joseph, Doctor)  *I tend to stick to the same thing over and over* (Kate, Doctor)  *We follow a protocol…this is what you do, and this is the frequency you give…I’m a firm believer that with…medications and drugs that we should be looking them up each time* (Sally, Doctor)  *I think that the hesitation on diaz is…because it’s a locked-up drug. You’re like, ‘Okay. Now I’ve got this escalated kid and I want to go find the keys, get the drug, find another nurse, check it out, come back, and who knows what’s going to happen in that five minutes I’m gone? The hard work we’ve done to get this kid to accept an oral medication, is it going to – are we going to still be all right?* (Patti, Nurse) |
|  | Medication dosing | *My strategy is to give much more [medication] than I think I should give…now if this kid should get five milligrams, I say, ‘That’s fine. This kid is getting 10 to 15.’…that doesn’t always make everyone happy…but if I’m going to give an IM, I don’t want to…just make then angry….I’d rather give them one injection…I know the side effects and we’re in a place we can deal with all the side effects* (Harry, Doctor)  *If it does need to be done, I’ve seen far more things go wrong…when an ineffective dose of a sedative is given…it doesn’t provide sedation…so when pursued, it should certainly be safe…it should be done at a sufficient dose* (Joseph, Doctor)  *Benzodiazepines are very safe drugs. They’re not going to cause harm to them. Usually, you just need to find the right dose. It’s sometimes even twice or three times what you might have expected…if they’re willing to take orals, then I would just usually continue…until they are settled* (Chris, Doctor) |
|  | Challenges of using medication in the pre-hospital setting | *With ambulance, our hands are slightly tied because our agitation/sedation guideline…has to be under consultant with the receiving hospital…hospitals aren’t really comfortable to let us sedate children in the field, a lot of times…I’ll give you [an] example of [a] girl…we’d be called by police…they would be holding the patient down because they would be so agitated, such a danger to themselves and others…I have to go ‘oh, I’m sorry, I just need to make a phone call to [redacted] hospital’…[the hospital] go ‘you can give them 2.5mg of midazolam.’ You go, ‘this patient is scream, thrashing and throwing fully grown men, I don’t think that’s going to work’…then I have to get a sharp out and sedate them, and then that’s not going to work, then I have to call them back and repeat the whole process again* (Chloe, Paramedic)  *We’re totally hamstrung by the fact that it’s now completely off the table to do anything without consulting the receiving hospital regardless of circumstance…I can think of a couple of cases where I’ve had an extremely agitated, let’s say 15-year-old who’s essentially the same size as me…and who’s violent, agitated and screaming…we get these recommendations for doses that we would deem to be inappropriate because it’s hard to pain the picture of how angry or violence a patient is when you’re not there* (John, Paramedic)  *One of the cops has been bitten. They’ve got three people holding him, they got his head against the wall so he doesn’t spit and bite on anyone…because he’s 15…we need to call up…I’m literally explaining…he’s got a [high agitation score] and he needs ketamine. This doctor, ‘you can’t use ketamine on children.’ I was just like ‘that’s completely incorrect’...he says ‘No. We never ever use ketamine. I’ve never seen someone who’s needed ketamine.’ I almost wanted to say ‘of course, you haven’t, because anyone who needs ketamine is brought by ambulance…you don’t bring a patient with [high agitation score] in your private vehicle* (Mark, Paramedic) |
| 2.5 Behavioral management plan |  | *We’ve had what we call care plans around for a long time…it takes a couple of minutes to access them…they’re fairly easy to read through…it’s got, in point form, what their main presentations are, some of the general de-escalation strategy…things to be talking about…it usually doesn’t talk about the drug or the dosages* (Peter, Paramedic)  *What we’re getting better at doing is…behaviour management plans for consumers…identifying what they like, what they dislike, what are their triggers…just so that we’re consistent of the way we treat consumers, particularly young people who regularly visit our health service* (Anthony, Mental Health Clinician)  *If you don’t know the person – one of things that I have seen…is they have a behavioral support profile…unfortunately, it is extremely lengthy…I just want to know…why we shouldn’t restrain this person, why we shouldn’t get this medication or how to work with this person… I need to know now, right now, what to do with this young person* (Karen, Mental Health Clinician) |
| 2.6 Physical and/or mechanical restraint |  | *Having appropriate restraints, first of all for these varied ages, I think that is the key…they worm around because they’re so small* (Nathan, Paramedic)  *If you chemically restrain someone, they also get physically restrained…they go hand-in-hand…in terms of physical restraints, I think our threshold’s probably lower because we’re in a moving vehicle…we’re often on highways…just pull over right then and there to get out of the ambulance, isn’t an option* (Mary, Paramedic)  *I would not keep a child physically restrained for an extended length of time…there’s no hard and fast…it’s if you’re thinking, okay, this person is still in a restraint, then, okay, this person is going to need to go to sleep* (Chris, Doctor)  *One girl, she had a history of trauma, borderline personality disorder, all of these things…she’d actually be in ED and mechanically restrained. She was restrained overnight and she was so traumatized…she refused to go back to hospital* (Beth, Mental Health Clinician) |
| 2.7 Differences in environment between the pre-hospital and ED settings |  | *I think working in a pre-hospital environment helps you to distill things a bit further, in that when you need to intervene here and now, in a pre-hospital environment, it requires…a more decisive, immediate approach because things can spiral more rapidly* (Joseph, Doctor)  *I’m not in a controlled environment. I don’t have all these resources available to me that are available in the hospital setting. I can’t employ all these different strategies or titrate medications* (Mary, Paramedic) |
| 3. Risk of harm | | |
| 3.1 Balance between patient and staff safety |  | *It was like…what is my threshold for feeling unsafe or being able to control the scene…but also balancing trying not to do future harm to [the patient]?...It was just really this internal dialogue about…how does [giving parental sedation] influence long-term. I know they discussed that there might be cognitive delays or impairments in the future associated with high-dose ketamine* (Mary, Paramedic)  *You’re trying to keep that young person safe, you’re trying to keep yourself safe as well, and your colleagues* (Pamela, Paramedic) |
| 3.3 Physical violence |  | *They are potentially high-risk people that can whip out a razor blade and stab you. We have to be careful with them. Our approach is probably being conservative to make sure that the staff are protected* (Kate, Doctor)  *They kicked off big time. They actually got a police gun out of a holster…fortunately the old copper was quite smart, there was nothing in it…it happened that fast* (Nathan, Paramedic)  *A kid at one stage threw a couch through the window…we don’t really have a proper, safe, low-stimulus environment* (Betty, Doctor)  *Plenty of the staff all the time are unfortunately getting assaulted* (Kate, Doctor)  *He was wielding an axe in community…everyone was very afraid of him* (Nicole, Doctor)  *There’s been some pretty severe things that have happened here. Computers being torn off the desk and broken, a knife has been pulled recently…I don’t believe any of those staff got followed up…probably because the timing was just not right at the time* (Hazel, Nurse)  *They will attempt to bite, and they just usually spit at you…this particular girl…she’s assaulted about 60 paramedics…I don’t think she actually ever gets in the ambulance because she’s too violence* (Chloe, Paramedic)  *I remember distinctly someone…asked me to get this patient a glass of water…I went to hand over the glass…and she grabbed it and threw it in my face…violence against staff – it’s still increasing. We have had nurses be injured to the point where they’ve been on leave for months, years as a result* (Annabelle, Nurse) |
| 3.4 Minimising or accepting violence |  | *I haven’t been physically injured, thankfully. I’ve been hit and slapped and things like that, but nothing that’s really hurt. I’m probably luck on that front* (Charlie, Nurse)  *I think it depends on the amount of exposure. If it’s something that doesn’t happen very often, the staff can be much more tolerant to verbal or even physical violence. If it’s something that’s very frequent, that’s the point where…the staff’s tolerance to that kind of behaviour has changed* (Julie, Doctor)  *I feel like you just get used to it, which is not the right way to look at it really, is it…I feel like it’s almost a little bit acceptable that they’re like that even though it’s not okay to ever get hit* (Harriet, Nurse)  *We had a young person in, and it was a young nurse who went in to see this patient, and it was a mental health patient that came in quite escalated…the young nurse got kicked in the face, and the senior-level nurse said, ‘that serves you right for putting yourself in that situation’* (Patti, Nurse)  *As hard as it is, I guess letting them abuse [you], they’ve just got verbal diarrhoea at that time anyway. Often, I don’t think they’re really necessarily purposefully trying to call you those names, it’s just they’re angry. I guess just letting them let off some steam* (Hazel, Nurse) |
| 4. Personal impact | | |
| 4.1 Emotional reactions |  | *The first word that comes to mind is challenging, mainly because of the emotional aspects that goes with looking after kids that come in with…acute behavioral disturbance…it feels as if there’s no one looking out for that child…it feels a little hopeless at times because you don’t feel like you’ve made…you may not have made much of a difference…you’ve been left with a little bit of, ‘I wish I could have done more’* (Annabelle, Nurse)  *It's just…very cognitively draining. You walk away from those jobs and you’re like, wow, I am exhausted and I don’t think I’ve even done a good job* (Mary, Paramedic)  *These jobs potentially sit with me longer than other jobs, as in they’re the ones that I go home and think about. I think the reason, primarily, is because there’s a sense of helplessness. I feel like we’re probably just one cog that contributes to this ongoing wheel for this young person…the service system…it’s falling short in meeting that young person’s needs…there’s this sense that…I’m contributing to this really traumatic journey in and out of services and health services* (Pamela, Paramedic)  *These are special cases because they’re a bit more emotive than probably our average job. You see these families that are just at breaking point…it can definitely be really emotionally draining* (John, Paramedic)  *Sometimes I would be absolutely petrified, very fearful that I was going to be assaulted* (Anthony, Mental Health Clinician)  *It’s disturbing at times how they do present and how they speak to you and some of the threats they might make. I don’t believe a lot of the threats, but you have to take them seriously…the most recent one where I did have the threat of, ‘I’m going to murder you – I’m going to kill you when I get out of this room’...this was a night duty…it’s so challenging to go home…go to bed with that in your brain* (Hazel, Nurse)  *I look back at situations…that sticks in my mind because I’m like, it just could have gone so much better and it just didn’t need to get to the point where we were manhandling a [child] and throwing him out the door when he needed help and he was saying he needed help* (Charlie, Nurse)  *It depends on my own bias I bring at the time. If I look at this patient and think, ‘you’re doing this on purpose’…I feel frustrated…for people that I think that they don’t really have control…over the way they’re behaving…I feel a degree of responsibility…and a degree of empathy…and a degree of sadness that whatever’s happened that has led them to this circumstance* (Harry, Doctor)  *They’re very emotive…they have this amazing way of getting under people’s skin…it can be very emotionally draining…there’s this transference of all this agitation* (Nicole, Doctor) |
| 4.2 Debriefing |  | *I feel like it happens – when we have a [cardiac] arrest, I guess we would debrief one, they don’t happen often and obviously it’s very stressful for staff involved. I feel like we have [a child with acute behavioral disturbance] most days and it’s always at the busiest time of the day…it is stressful, especially if someone gets assaulted or hurt…we don’t…do a debrief* (Harriet, Nurse)  *We never debrief. I feel like I’ve been in some pretty nasty Code Blacks and stuff where you…can’t believe that just happened. We never debrief, it’s awful* (Hazel, Nurse)  *We don’t really talk about [acute behavioral disturbance] a lot. We’re always talking about the…cardiac arrest, and the big traumas…but not the mental health…I’ve touched base with [the paramedics involved]…and they’ve actually reached out…and said, ‘I can’t believe what we did the other night.’ It obviously impacts them* (Nathan, Paramedic)  *I think regular clinical supervision is helpful just to debrief…also that reflective practice discussing treatment plans…that was exceptionally helpful* (Anthony, Mental Health Clinician)  *I really do think that we would benefit from debriefing in those situations, particularly when we’ve had to, say, restrain a 16-year-old…we never want to restrain people…I think a debrief would really, really benefit us in improving our management* (Charlie, Nurse) |
| 4.3 Rationalising or humanising the presentation |  | *I always take that approach with remembering that these kids come from unimaginable trauma for the most part…it’s always good to have that in the back of your mind* (Charlie, Nurse)  *Remembering that there’s a whole story behind that child and separating the child from the behaviour is really important because if you start labeling the child, then you’re going to have a lot more difficultly in wanting to work with the child* (Beth, Mental Health Clinician)  *I see this badly behaved teenager but if you get the story, they’re abused by their father, they’ve been in care all their lives…you come in with a different – it’s easy then to walk in with your professional hat on rather than this frustration with this badly behaved teenager* (Nicole, Doctor)  *If you look at a child that is a two-year-old child that’s difficult – a work of breather and they’re irritable and they’re cranky…it’s not the child. They feel rubbish. Some of these kids that have these outbursts and it’s not maybe necessarily the human, but a reaction to their disease* (Patti, Nurse)  *This behaviour is in response to something. All behavior is meaningful, and that we don’t know what it is, it’s on us to find out…it’s not the fact that they’re naughty children. It doesn’t justify the behaviour but what is this in response to, is a question I think we need to constantly be asking ourselves* (Carmel, Mental Health Clinician) |
| 5. Workflow, resource and bystander impacts | | |
| 5.1 Ambulance ramping |  | *I had a few instances where we had transported [a behavioral disturbance patient], and then we just ramped….as you know, sedation doesn’t last for ages. Then you’ve got a patient that’s becoming heightened in an ED…they don’t have any beds…you’ve got other paramedics looking to you and sometimes being like, what are you doing to control your patient?...these can be very long jobs when you’re met with that ramping…it’s not the hospitals fault. It’s the state of healthcare. It does make it hard* (Mary, Paramedic)  *It's the ability to be able to streamline them through the system, so that they’re not sitting, waiting for ages, and then you’re getting escalation so that they need further sedation or they need security or they need tying down or they…jump off the stretcher and they leave the facility* (Peter, Paramedic)  *He was...on my stretcher for four and a half hours…that was just one of those grinding, escalating things. It was just…what was going to tick him off at the end…ramping, it’s been horrific. It’s been soul destroying a lot of the time and you turn around and I’ve had people go, I just can’t do anymore* (Nathan, Paramedic) |
| 5.2 Staff time allocation |  | *The reality is…I’m the doctor in charge of the emergency department, and if an emergency comes, you’re going to be left hanging…and the problem is, then the department gets completely constipated…there’s this half-sorted stuff* (Paul, Doctor)  *It does disrupt the running of the department…if the consultant is caught up in a single case…for half an hour…an hour…then that’s probably a good 20-odd people that have moved through that you haven’t done anything with…and if someone is sedated and in resus, then that’s, again, a significant nursing load* (Chris, Doctor)  *If I go in to get involved in that, then I’m going to neglect the rest of the emergency department because that’s usually what that presentation needs to get the endpoint for that patient that they need. I might sit there for an hour, if required, and just let the place go to shit* (Sam, Doctor)  *Every time you sit there and take longer to do things, it’s a nurse that’s a one-on-one off the floor…it has a massive knock-on-effect…it’s quite tricky* (Harriet, Nurse)  *Sometimes, you can end up there for hours and hours…a big issue every day at the moment is job times exploding and therefore crews not being available for other calls…unique to our service compared to a hospital where the patients come to us, we have to be able to come to patients. We sometimes have to think of the broader community in the sense of how much time do we spend here* (Mark, Paramedic) |
| 5.3 Effect on bystanders in the emergency department |  | *A thing we worry a lot about here is…we have a situation where someone escalates and they’re running around and aggressive and maybe verbally aggressive and violent, they’re in really close proximity to other kids, other children, and so you always are a bit scared about the repercussions of that and what could potentially happen* (Annabelle, Nurse)  *If they’re very loud and threatening, again, you’ve got other patients…and they get scared – like we get complaints…they’re so afraid…because there was all this shouting. It’s just impossible* (Nicole, Doctor)  *Families and all the other staff can’t help but hear…it’s really difficult when someone is going ballistic to hide that from everyone. Naturally just draws everyone’s attention, it’s awful* (Hazel, Nurse)  *The other thing we need to be aware of…is the bystander effect. One young person might escalate for a specific reason and then another young person might chime in…then trying to de-escalate two people who are agitated and aggressive…can make things considerably more difficult* (Anthony, Mental Health Clinician) |
| 5.4 Lack of timely and appropriate disposition |  | *[You’ve got a] pediatrician saying, ‘well, I’m a pediatrician, but a 15-year-old who is 120 kilos and going absolutely nuts, I’m not looking after them’ and mental health saying, ‘well, it sounds that’s a health problem because they’ve got intellectual disability or autism’ so they stay [in the ED]…no one’s taking responsibility…ED is saying ‘can you get them out of my ED. I’ve done my bit….can someone sort them out? Mom doesn’t want to take him home.’ We need a better system….the elephant in the room is it’s difficult…the autism spectrum disorder, intellectual disability group fall through the cracks because no one wants to take responsibility* (Ben, Doctor)  *The negotiation about where next is also a very trick one, because if you have someone who is agitated, the wards…are not really well set up for someone who is likely to become aggressive and agitated…often, they need to go to the psychiatry ward, even though they’re not really a psychiatric patient, just because the behavioral management needs to be done…sometimes you get children…particularly the autistic spectrum, ones that are not safe to go to the ward, who don’t need to go to psychiatry…they’ve spent several days in the emergency department trying to find an appropriate place…it does get very complex* (Chris, Doctor)  *Our pediatric ward currently is not very well set up for adolescents who have agitation, and then they’ll get [stuck] down in the ED because they’re not appropriate for our mental health unit as well* (Sally, Doctor) |
| 5.5 Community support or follow up |  | *There’s no quick fix…but you know that person’s going to need so much more when they leave here for proper therapy…does it ever get done? I don’t know. Sometimes we see them again and similar situations happen again* (Hazel, Nurse)  *Families [tell] me that they feel [community support] is not as helpful…once the funds are exhausted, where do they go from there. Sometimes the emergency department is their last course of action* (Anthony, Mental Health Clinician)  *Child protection units are often…under-resourced as well…often they are like ‘oh, we’ll get someone over there tomorrow.’* (Nicole, Doctor)  *That’d be the key, obviously, less hospital presentations. What’s the safest way? How do we manage them and leave them at home…is there some other way that they can be managed better?* (Nathan, Paramedic)  *From our perspective…it’s that pattern. This is what we do, we go, we treat, we transfer the young person back into the community. We go, we treat, we transfer…despite all attempts to try and coordinate…we don’t seem to come up with effective ways to support that young person to not have to navigate that cycle* (Pamela, Paramedic)  *I think the thing with me that I kind of – I think…all the support places, they say, ‘oh, yes, and if this happens phone 000 and go to the ED.’ People just view us as, go to the ED and it’ll fix it, and I get it. We’re open 24/7, there’s always people here. You can be expected to be safe here, but also it’s like there’s still nothing we can do* (Cassandra, Mental Health Clinician) |
