# Supplementary material for: Qualitative Exploration of Health Care Professionals’ Experiences Caring for Young People With Acute Severe Behavioral Disturbance in the Acute Care Setting
Source: J Am Coll Emerg Physicians Open. 2025 Jan 13;6(1):100030. doi: 10.1016/j.acepjo.2024.100030 (PMC11852700; doi:10.1016/j.acepjo.2024.100030)
Supplement: Supplementary Table 1 [file mmc3.docx]

Supplementary Table 1: Emergency Physician Experience

| **Contributing factors and de-escalation strategies** |
| --- |
| *The most recent child…came in as a 17-year-old who is in that horrible prodrome of schizophreniform illness…his violence, which is dissociative, it’s almost like a guarded dissociative state where he seemed to randomly just go completely insane and want to hurt anyone that was near him and smash everything* (Sam, Doctor) |
| *The kids with autism…or other intellectual disability who aggression is their way of expressing frustration…I think the worst behavioral patients I’ve looked after fit into this category…[the parents] get to the point where they just say ‘my kid’s now 15 and he’s too big, I can’t restrain him…he’s breaking all my walls* (Harry, Doctor) |
| *You need to know how to be able to talk to them. Doctors who are very doctor-like and very prim and proper and talk the medical language, they’re not going to get through to a 15-year-old that’s going off* (Ben, Doctor) |
| *I tend to be pretty relaxed…I sit down and I usually just have a social chat if I can first to get a feel for what’s going on…try and make them as comfortable as I can* (Sally, Doctor) |
| *I sit on the end of the bed. I introduce myself. I tell them I’m here to help them with the intent of engaging in a therapeutic alliance with them. I then ask them something about themselves. Usually, I’m asking them about hobbies…getting to know a little bit about them works sometimes* (Paul Doctor) |
| *Probably, oh, about 50% of the time I suspect we’re successful in just calming everything down without resorting to pharmacological methods* (Julie, Doctor) |
| **Medication management** |
| *Quantifying when there is in fact an urgent risk, is the most useful frame I find…it’s the minority of cases in kids who do need chemical sedation but when they do, it shouldn’t be deferred* (Joseph, Doctor) |
| *I’d certainly give them the opportunity to take something [oral] first. The phrasing is important. ‘Look, it’s just to take the edge off things, not to knock you out. It’s just to make you feel less agitated so we can have a proper talk in a little while.’* (Chris, Doctor) |
| *I tend to stick to the same thing over and over* (Kate, Doctor) |
| *[Drug choice is] definitely variable depending on the child, the etiology, and circumstance. …there’s so many different encounters or flavours of encounters* (Joseph, Doctor) |
| *I have a lot more experience with ketamine…I try to do things that I know that I’m comfortable managing. I’ve given lots of kids ketamine. If I had an acutely disturbed kid…I’d be reasonably comfortable giving them an IM dose of ketamine* (Betty, Doctor) |
| *My strategy is to give much more [medication] than I think I should give…now if this kid should get five milligrams, I say, ‘That’s fine. This kid is getting 10 to 15.’…that doesn’t always make everyone happy…but if I’m going to give an IM, I don’t want to…just make them angry….I’d rather give them one injection…I know the side effects and we’re in a place we can deal with all the side effects* (Harry, Doctor) |
| **Occupational violence and aggression, emotional impact of care and debriefing** |
| *They are potentially high-risk people that can whip out a razor blade and stab you. We have to be careful with them. Our approach is probably being conservative to make sure that the staff are protected* (Kate, Doctor) |
| *A kid at one stage threw a couch through the window* (Betty, Doctor) |
| *He was wielding an axe in community…everyone was very afraid of him* (Nicole, Doctor) |
| *It depends on my own bias I bring at the time. If I look at this patient and think, ‘you’re doing this on purpose’…I feel frustrated…for people that I think that they don’t really have control…over the way they’re behaving…I feel a degree of responsibility…and a degree of empathy…and a degree of sadness that whatever’s happened that has led them to this circumstance* (Harry, Doctor) |
| **Resource limitations, disposition and follow up** |
| *If I go in to get involved in [the patient presenting with acute severe behavioral disturbance], then I’m going to neglect the rest of the emergency department because that’s usually what that presentation needs to get the endpoint for that patient that they need. I might sit there for an hour, if required, and just let the place go to shit* (Sam, Doctor) |
| *The negotiation about where next is also a very tricky one, because if you have someone who is agitated, the wards…are not really well set up for someone who is likely to become aggressive and agitated…often, they need to go to the psychiatry ward, even though they’re not really a psychiatric patient, just because the behavioral management needs to be done…sometimes you get children…particularly the autistic spectrum ones that are not safe to go to the ward, who don’t need to go to psychiatry…they’ve spent several days in the emergency department trying to find an appropriate place…it does get very complex* (Chris, Doctor) |
